# Hematological, biochemical, and serological parameters of experimentally infected rabbits with *Trichinella nativa* and *Trichinella spiralis* for early identification of trichinellosis

**DOI:** 10.14202/vetworld.2022.2285-2292

**Published:** 2022-09-24

**Authors:** Orken S. Akibekov, Alfiya S. Syzdykova, Lyudmila A. Lider, Aibek Kh. Zhumalin, Zhasulan K. Baibolin, Fariza S. Zhagipar, Zhannara Zh. Akanova, Ainur A. Ibzhanova, Aissarat M. Gajimuradova

**Affiliations:** Department of Microbiology and Biotechnology, Faculty of Veterinary and Livestock Technology, Saken Seifullin Kazakh Agrotechnical University, 62 Zhenis Avenue, Nur-Sultan 010000, Kazakhstan

**Keywords:** diagnostics, experimental infection, rabbits, *Trichinella nativa*, *Trichinella spiralis*, trichinellosis

## Abstract

**Background and Aim:**

Trichinellosis remains a dangerous disease for humans and animals, which can lead to a lethal outcome. The study of specific body reactions in response to invasion by different types of *Trichinella* can help in the early diagnosis of the disease. This study aimed to investigate the hematological, biochemical, and serological characteristics of rabbits experimentally infected with trichinellosis, as well as the possibility of using changes in these parameters at various disease stages for early hematological, biochemical, and serological diagnosis of trichinellosis.

**Materials and Methods:**

Three groups of rabbits were orally infected with *Trichinella nativa* and *Trichinella spiralis* derived from encysted *T. spirtalis* larvae in pork muscle samples. The first and second groups were infected with *T. nativa* and *T. spiralis*, respectively, while the third group served as control by receiving a physiological solution. An ADVIA 2120i automatic hematology analyzer with a blood smear staining module was used to determine the hematological parameters of rabbits. Antigens were used in an enzyme-linked immunosorbent assay (ELISA) to detect antibodies in the sera of infected rabbits that were supernatants containing excretory-secretory antigens (ES-Ag) and somatic antigen (S-Ag).

**Results:**

The detection of biochemical responses to the invasion of *T. nativa* and *T. spiralis* isolates was detected and hematological parameters were featured in two cases. *Trichinella nativa* increased the number of erythrocytes, neutrophils, eosinophils, monocytes, basophils, and thrombocytes on day 7 in rabbits. Creatine kinase (CK) is regarded as the most important indicator for the early detection of parasite invasion. Blood biochemistry showed no active response to *T. spiralis* infection. However, counts of erythrocytes, neutrophils, lymphocytes, and CK rose significantly. In both color indicators, the number of thrombocytes decreased. Enzyme-linked immunosorbent assay with ES-Ag and S-Ag of these isolates demonstrated the ability to detect antibodies as early as 7 days after infection, with a significant increase in the marker up to 70 days.

**Conclusion:**

On the 7^th^ day after infection, blood tests of infected animals revealed CK-N-acetyl-cysteine (18.2%) and neutrophils (43%) when infected with *T. nativa* and neutrophils (26.7%) and lymphocytes (20%) when infected with *T. spiralis*. These indicators may serve as specific parameters for the early detection of *Trichinella* spp. invasion.

## Introduction

Trichinellosis is a serious zoonotic disease caused by *Trichinella* nematodes. People become infected by consuming raw or undercooked meat containing *Trichinella* spp. larvae, particularly from wild animals such as bears, boars, and domesticated animals such as pets (pigs and dogs) [1]. According to the Centers for Disease Control and Prevention (USA), approximately 10,000 cases of trichinellosis are reported annually worldwide, primarily due to the consumption of pork and, more frequently, wild animal (bear) meat [2]. In 2019, 96 cases were detected in 12 EU member states, with Bulgaria, Italy, and Spain accounting for 79.2% of cases. The overall incidence rate is 0.02 per 100,000 individuals [3]. Germany had 0.01 cases of trichinellosis per 100,000 population in 2007 [4], according to data, China [5], Thailand [6], and Vietnam [7] are endemic among Asian nations.

Approximately 0.58 cases of trichinellosis per 100,000 people are reported annually in Kazakhstan, but these numbers are inaccurate. According to Abdybekova *et al*. [8] there was a single case of trichinellosis among the country’s population in 2014.

After eating infected meat, *Trichinella* larvae are released from their capsules, enter the upper portion of the small intestine, and mature into adult worms. This causes the development of trichinellosis. After 2–3 weeks, fertilized females produce approximately 1500 larvae, which migrate through the circulatory and lymphatic systems, penetrate skeletal muscle, and encapsulate (muscular or acute phase). The acute phase is characterized by an inflammatory and allergic response following the larval invasion of muscle [9].

Human trichinellosis is challenging to diagnose clinically because its clinical manifestations are nonspecific. At present, a definitive diagnosis of human trichinellosis can be made by detecting larvae in a muscle biopsy specimen or specific antibodies against *Trichinella* immunoglobulin G (IgG) [10], but parasitological examination of biopsy specimens is insufficiently sensitive to detect *Trichinella* larvae in mild infections and early stages of infection.

Enzyme-linked immunosorbent assay (ELISA) with excretory-secretory antigen (ES-Ag) is the most common serological test for trichinellosis diagnosis and is recommended by the International Commission on Trichinellosis. The main disadvantage of detecting anti-*T*. *spiralis* Ig antibodies using commercial ELISA tests against ES antigens is the high frequency of false-negative results at an early infection stage [11]. Furthermore, these false-negative results may occur because most *Trichinella* spp. are specific to a certain stage of *Trichinella* development. Consequently, parasite-induced antibodies do not recognize ES antigens during the intestinal phase. Multiple studies have demonstrated that the maximum detection rate of all (100%) *Trichinella* IgG antibodies is not reached until 1–3 months following human infection with the parasite [12].

There are no definitive rabbit hematology studies in the scientific literature. Diseases are typically diagnosed by drawing blood and analyzing it for the presence and nature of an inflammatory process in the body. There is a clear protocol for determining bacterial or fungal infections through blood tests in the presence of bacterial or fungal infections. In the case of parasitic invasion, such protocols do not exist. Therefore, it is urgent to develop new methods for the early diagnosis of parasite infestation using blood chemistry at 7, 14, 31, 45, and 70 days after *Trichinella* infection. This paper presents the results of biochemical and serological studies conducted on the blood of rabbits experimentally infected with isolates of *Trichinella nativa* and *Trichinella spiralis*, as well as their potential application in the early diagnosis of trichinellosis.

This study examined hematological, biochemical, and serological parameters in response to invasion by *T. nativa* and *T. spiralis* isolates to aid in the early diagnosis of trichinellosis.

## Materials and Methods

### Ethical approval

The study was approved by the Animal Ethics Committee, Faculty of Veterinary and Livestock Technology, S. Seifullin Kazakh Agrotechnical University (KATU), and performed in accordance with the Guidelines for Accommodation and Care of Animals: Species-specific provisions for laboratory rodents and rabbits (Interstate Standard, GOST 33216-2014). All protocols were performed in accordance with the International Guiding Principles for Biomedical Research Involving Animals [13].

### Study period and location

The study was conducted from March to December 2021. The laboratory animals used in the study consisted of 12 male Soviet chinchilla breed of rabbits aged 7–8 months with a live weight of 4400–4600 g. The rabbits were maintained under appropriate hygienic conditions in the vivarium of KATU, Nur-Sultan, Kazakhstan.

### Infection of animals

We used muscle tissue samples of spontaneously infected wild animals and muscle tissue samples of pigs experimentally infested with *Trichinella*, which were provided to us by a specialist in the Department of Diagnostics, Genetics and Characterization of the Pathogen of the Reference Center for Risk Assessment (BfR) Berlin (Germany), Dr. Anne Mayer-Scholl. All samples of naturally infected wild animals and experimentally infected pigs. Polymerase chain reaction analysis was used to identify all *Trichinella* larvae samples to the species level through five specific pairs of primers for internal transcribed spacers 1 (ITS1), ITS2, and expansion segment V sites [14].

For this experiment, nine rabbits aged 7–8 months (immature immune system and approximately same weight) were selected and dewormed (each group by three repetitions). Based on the analogy principle, three experimental animal groups were then created. The trichinellosis-causing agents *T. nativa* and *T. spiralis* were administered at doses ranging from 2500 to 3000 larvae per head to the three rabbits comprising Groups 1 and 2. The *Trichinella* larvae-containing “formula” was injected per os into the animals using a disposable pipette. Three non-infected animals serving as controls (group 3) were administered 5 mL of saline orally. In addition to clinical observations, monthly floor scale weights and body temperature measurements were performed on rabbits. Seventy days were spent conducting experimental studies.

On day 70, animals infected with *T. nativa* and *T. spiralis* were euthanized for post-mortem autopsy using sequential intramuscular injections of 1.5 mg/kg xylazine and intravenous injections of 7.5 mg/kg anesto­fol^®^ (Russia). The carcasses of the animals were examined for the presence of parasites in accordance with the World Health Organization/World Organization for Animal Health recommendations [15] after the muscles were surgically opened.

Blood was drawn from rabbits before infection, on days 7, 14, 31, 45, and 70 after infection for hematological, biochemical, and serological analyses. Using a BD Vacutainer (Guangzhou Improve Medical Instruments Co., Ltd, China), vacuum system, blood was drawn from the marginal vein of the ear and the saphenous vein of the forearm.

### Hematological studies

Subsequently, hematological studies were conducted using an ADVIA 2120i automatic hematological analyzer with a blood smear staining module (Siemens Helthcare Diagnostics Inc., USA). In Panchenkov capillary tubes, the erythrocyte sedimentation rate was determined using the conventional method.

### Serological analysis

The serological analysis of rabbits was conducted using polystyrene 96-well flat-bottom ELISA plates (Alto, Italy) and the standard indirect ELISA protocol. The wells of the tablet were sensitized overnight at 4°C with parasite antigens at a concentration of 0.010 mg/mL. Next, beginning with a 1:100 dilution, antisera samples of 0.1 mL were titrated into the wells of the plate. The plates were then incubated for 60 min at 37°C. Subsequently, causing an antispecies conjugate (Jackson Immuno Research, USA) and its substrate, orthophenylenediamine (OPD), the immune complex was detected (Sigma, USA). The plate was washed 3 times with PBS and three times with PBS-Tween to separate the unbound reaction components from the solid phase. Positive ELISA results were determined if the optical density (OD) value of the well containing antiserum was at least 2 times greater than the OD value of the control well containing pre-infected rabbit serum.

### Statistical analysis

Standard statistical analysis of hematological parameters and ELISA results was conducted using the t-student test (t) and the p-values (p).

## Results and Discussion

### Experimental infection of rabbits with *Trichinella spp.* larvae obtained from wild animals

From day 7 to day 31 of the experiment, a slight loss of appetite was observed in two rabbits while observing infected animals. On the 14^th^ day of the experiment, one rabbit administered 3000 *T. spiralis Trichinella* larvae exhibited hyperthermia. This animal’s body temperature remained within the acceptable range from day 7 to day 31. Animals in the experimental group gained significantly less weight than those in the control group. On day 70, rabbits administered between 2500 and 3000 *Trichinella* larvae weighed 4.4, 4.3, 4.0, and 3.95 kg compared with 4.6 kg in the control group (p ≥ 0.05).

### Hematological blood tests on *Trichinella nativa*-infected rabbits

On day 7, biochemical studies of the blood of infected rabbits revealed significant changes in nine blood parameters. The major inflammatory and allergic reaction markers are neutrophils, eosinophils, monocytes, and basophils. These markers exhibited respective changes of 42.6%, 52.5%, 93.8%, and 20%. In the later stages, encapsulating nurse cells contain an abundance of neutrophils [16, 17]. Data on biochemical parameters of blood of experimentally infected animals are presented in Tables-1 and 2.

**Table-1:** Dynamics of hematological and biochemical parameters of rabbits infected with *T. nativa*.

Parameters	Average parameters before infection	Days after experimental infection of rabbits				

		7 DPI	14 DPI	31 DPI	45 DPI	70 DPI
Hb, g/L	115 ± 0.02	116 ± 0.06	119 ± 0.03	120 ± 0.03	126 ± 0.02	127 ± 0.06
Erythrocytes, 10^12^/L	4.87 ± 0.07	6.27 ± 0.10	5.32 ± 0.18	5.47 ± 0.02	6.47 ± 0.03	6.83 ± 0.10
Color indicator	0.97 ± 0.06	0.6 ± 0.13	0.68 ± 0.01	0.77 ± 0.03	0.67 ± 0.01	0.65 ± 0.08
Hematocrit, %	39.4 ± 0.03	37.27 ± 0.13	39.01 ± 0.06	37.48 ± 0.01	40.36 ± 0.01	36.5 ± 0.07
Thrombocytes, 10^9^/L	378.3 ± 0.04	279.1 ± 0.01	296.9 ± 0.04	298.0 ± 0.02	295.8 ± 0.03	312 ± 0.02
Leukocytes, 10^9^/L	7.47 ± 0.06	6.57 ± 0.01	6.14 ± 0.02	7.56 ± 0.02	8.55 ± 0.04	6.5 ± 0.14
Neutrophils,%	54.2 ± 0.06	77.5 ± 0.01	79.0 ± 0.02	77.46 ± 0.01	79.3 ± 0.07	79.7 ± 0.05
Eosinophils,%	1.6 ± 0.16	2.53 ± 0.04	2.61 ± 0.05	2.6 ± 0.16	2.31 ± 0.02	4.0 ± 0.10
Basophils,%	1.8 ± 0.04	2.11 ± 0.07	2.19 ± 0.01	2.3 ± 0.04	2.29 ± 0.02	2.23 ± 0.05
Monocytes	4.54 ± 0.03	8.8 ± 0.03	5.6 ± 0.09	9.6 ± 0.02	5.8 ± 0.15	4.3 ± 0.05
Lymphocytes	7.57 ± 0.01	7.5 ± 0.04	7.5 ± 0.11	8.14 ± 0.01	9.4 ± 0.08	12 ± 0.02
ESR, mm/h (according to Panchenkov)	2.0 ± 0.02	1.8 ± 0.05	2.0 ± 0.13	1.9 ± 0.04	2.1 ± 0.14	2.1 ± 0.21
Creatine kinase CK-NAC, U/L	772 ± 0.01	911.4 ± 0.01	929.3 ± 0.01	1358.7 ± 0.02	2220.4 ± 0.02	2243.3 ± 0.12
Immunoglobulin M, g/L	1.3 ± 0.13	0.92 ± 0.01	0.92 ± 0.01	0.88 ± 0.02	0.8 ± 0.05	1.1 ± 0.24
Immunoglobulin G, g/L	1.07 ± 0.15	1.3 ± 0.05	1.35 ± 0.02	1.36 ± 0.04	1.43 ± 0.06	1.1 ± 0.01

*T. native=Trichinella native*, CK-NAC=Creatine kinase-N-acetyl-cysteine, ESR=Erythrocyte sedimentation rate, Hb=Hemoglobin, DPI=Days post-infection

**Table-2:** Dynamics of hematological and biochemical parameters of rabbits infected with *T. spiralis*.

Parameters	Average parameters before infection	Days after experimental infection of rabbits				

		7 DPI	14 DPI	31 DPI	45 DPI	70 DPI
Hb, g/l	95.4 ± 0.08	96.3 ± 0.06	97.6 ± 0.08	95.7 ± 0.05	97.6 ± 0.07	96.6 ± 0.03
Erythrocytes, 10^12^/L	4.85 ± 0.05	5.16 ± 0.01	5.74 ± 0.05	5.66 ± 0.08	6.18 ± 0.24	5.92 ± 0.04
Color indicator	0.87 ± 0.05	0.65 ± 0.04	0.65 ± 0.11	0.78 ± 0.09	0.67 ± 0.01	0.69 ± 0.02
Hematocrit, %	40.16 ± 0.02	37.51 ± 0.03	43.23 ± 0.05	43.74 ± 0.06	40.43 ± 0.02	50.21 ± 0.03
Thrombocytes, 10^9^/L	355.5 ± 0.03	313 ± 0.11	295.0 ± 0.08	279.6 ± 0.08	291.1 ± 0.08	297 ± 0.03
Leukocytes, 10^9^/L	7.39 ± 0.04	6.36 ± 0.04	6.19 ± 0.06	7.57 ± 0.03	8.57 ± 0.01	6.5 ± 0.0
Neutrophils,%	7.59 ± 0.03	9.62 ± 0.01	9.62 ± 0.01	10.60 ± 0.01	9.87 ± 0.04	9.16 ± 0.01
Eosinophils,%	5.6 ± 0.04	5.8 ± 0.02	5.75 ± 0.04	5.86 ± 0.02	6.07 ± 0.01	5.97 ± 0.02
Basophils,%	0.2 ± 0.11	0.4 ± 0.16	0.1 ± 0.16	0.02 ± 0.04	0.04 ± 0.03	0.02 ± 0.04
Monocytes	8.06 ± 0.01	8.7 ± 0.04	8.7 ± 0.07	8.5 ± 0.21	8.8 ± 0.08	8.7 ± 0.02
Lymphocytes	47.46 ± 0.01	56.33 ± 0.41	52.51 ± 0.02	42.65 ± 0.05	47.33 ± 0.05	43.96 ± 0.02
ESR, mm/h (according to Panchenkov)	2.0 ± 0.04	2.0 ± 0.07	2.0 ± 0.0	1.97 ± 0.04	1.97 ± 0.04	1.97 ± 0.04
Creatine kinase CK-NAC, U/L	919.1 ± 0.05	874.6 ± 0.04	889.7 ± 0.09	911.6 ± 0.01	869.4 ± 0.02	902.7 ± 0.01
Immunoglobulin M, g/L	0.88 ± 0.09	0.87 ± 0.09	0.81 ± 0.10	0.86 ± 0.08	0.92 ± 0.04	0.89 ± 0.03
Immunoglobulin G, g/L	2.16 ± 0.02	2.02 ± 0.01	1.84 ± 0.04	2.32 ± 0.04	2.40 ± 0.01	2.08 ± 0.02

*T. spiralis=Trichinella spiralis*, CK-NAC=Creatine kinase-N-acetyl-cysteine, ESR=Erythrocyte sedimentation rate, Hb=Hemoglobin, DPI=Days post-infection

An increase in eosinophils and basophils directly indicates the onset of a primary-allergic reaction in the body on the 1^st^ day following infection. When they enter the body, *T. nativa* and *T. spiralis* cause a severe allergic reaction [18]. The release of histamines by basophil cells is responsible for the occurrence of allergic reactions. In the case of *T. nativa* invasion, both indicators remained elevated during the study. Moreover, the eosinophil index increases 2.5 times by day 70 relative to the control index.

In addition to the clinical manifestations of invasion during *T. spiralis* infection and its early diagnosis, it is possible to examine the blood’s neutrophil count. This study demonstrates that, compared to the control, this marker is elevated throughout the 70-day experiment. The presence of neutrophils and monocytes in the infiltrates of encapsulating nurse cells [19] suggests that an isolated increase in neutrophils on day 31 is indicative of capsule formation.

**Figure-1:**
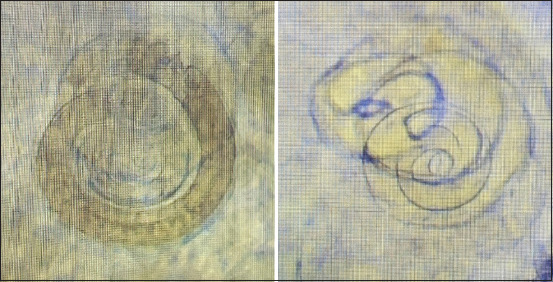
Larvae of *Trichinella nativa* and *Trichinella spiralis* in muscle tissue of rabbits under compression (100×) (original).

On day 70 after infection, rabbits infected with encapsulating species of *Trichinella* were euthanized, and sections of muscle tissue and diaphragm were examined parasitologically. Despite the rabbits’ active response to *T. nativa* invasion, its index was significantly lower than that of infection with *T. spiralis*: 166 ± 22.6, 730.6 ± 89.5, respectively (Figure-1). The nature and characteristics of encapsulating species of *Trichinella* larvae distribution in the striated muscles of rabbits revealed muscle tropism localization of these helminths in particular groups of skeletal muscles (Table-3). The muscles of the head (61.6 ± 51.3 and 197.3 ± 176.3), forelimbs (26.4 ± 21.5 and 143.5 ± 127.2), and chest walls (32.6 ± 27.3 and 137.8 ± 121.2) had the highest levels of invasion intensity in *T. nativa* and *T. spiralis*, respectively.

**Table-3:** The content of *T. larvae* in 1 g of muscle in experimentally infested rabbits.

S. No.	Muscle groups	Type of pathogen, dose of infection, Trichinella larvae per head	

		*T. nativa*	*T. spiralis*
		2500	3000
1	Muscles of the head (masseter, root of the tongue, esophagus)	61.6 ± 51.3	197.3 ± 176.3
2	Muscles of the shoulder girdle (brachiocephalic, trapezius, serratus and pectoral muscles, latissimus dorsi)	15.3 ± 11.0	74.6 ± 62.1
3	Muscles of the thoracic limb (deltoid, infraspinatus, supraspinous and prelochal muscles, muscles of the shoulder and wrist)	26.4 ± 21.5	143.5 ± 127.2
4	Muscles of the pelvic limb (semitendinosus, semimembranosus, adductor, biceps and quadriceps femoris, gastrocnemius muscle group)	19.1 ± 15.0	129.1 ± 102.4
5	Muscles of the spinal column (longissimus dorsi, psoas, neck)	6.0 ± 5.1	27.2 ± 23.6
6	Muscles of the chest wall (intercostal muscles, crura, diaphragm)	32.6 ± 27.3	137.8 ± 121.2
7	Muscles of the abdominal walls (oblique and transverse abdominal muscles)	5.0 ± 4.8	21.1 ± 16.3
Mean value, M ± m		23.7 ± 21.1	104.3 ± 101.8

*T. native=Trichinella native,T. spiralis=Trichinella spiralis, T. larvae=Trichinella larvae,* P/P ≥ 2 – positive result, P/P ≤ 2 – negative result.

According to Pozio *et al*. [20], this result is not associated with differences in adult reproductive capacity. Regarding infectiousness, Skrzypek and Nowakowski [21] infected rabbits with a single dose of 90 *T. spiralis* invasive larvae; 60 days after infection, masseter muscle tropism was observed. The trichinellosis larvae infestation was greatest in the antebrachii and diaphragm. The *T. spiralis* infectivity in the other examined muscles ranged from 1.08 to 17.13 times lower than that of the masseter muscle. Infectivity in muscles is directly proportional to the larval dose. According to the obtained data, the intensity of the distribution of encapsulated *Trichinella* forms is consistent. According to our findings, larvae invaded the head, pelvic limb, and chest wall muscles the least. A medium invasion was observed in the chest and pelvic limb muscles.

**Figure-2:**
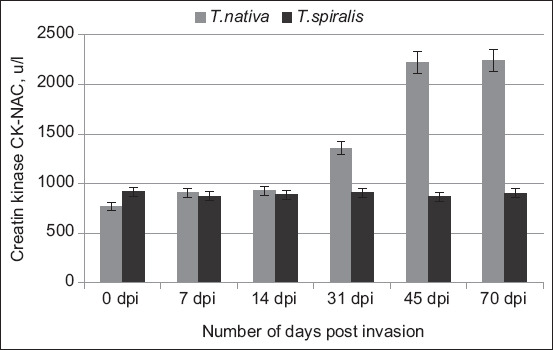
Dynamics of creatine kinase-N-acetyl-cysteine level with *Trichinella nativa* and *Trichinella spiralis* invasion.

Leukocyte levels fluctuated from day 7 to day 70 post-infestation with *T. nativa*, with a peak on day 45 that was 16% higher than the initial value. Unlike other forms of infection, this indicator is less informative. According to the leukocyte formula, effective diagnosis is possible at the earliest signs of helminth infection, when helminths can pass through the intestinal phase and initiate partial muscle penetration. During the 1^st^ week, creatine kinase-N-acetyl-cysteine (CK-NAC) levels increased by 18.2%. The results of biochemical studies conducted on rabbits infected with *T. nativa* isolate indicate that the process of parasite invasion into muscles can be observed from day 7 to day 70, as evidenced by an increase from 18.2% to 191% in the level of CK-NAC (Figure-2).

NAC-activated CK is an important marker for skeletal muscle degradation. This marker is essential during myocardial infarction, myopathy, dystrophy, and brain tissue changes. However, none of the existing diagnostic recommendations for parasitic infections require testing for this marker. A comparison of CK rates between the two parasite species and the immune response they elicit reveals that this rate increases significantly during the 1^st^ week of *T. nativa* invasion. However, *T. spiralis* causes this indicator to decrease by 7 days post-infection (DPI) by 4.8%, then gradually increase to 3.2% (14 DPI), 0.8% (31 DPI). The results may suggest a difference in the level of muscle invasion between the two species, as well as a difference in the immune response to various sources of infection.

*Trichinella nativa* invasion is accompanied by a noticeable long-term response in terms of blood biochemistry. This may account for the lack of specificity of this parasite species for rabbits. Thus, based on an analysis of muscle group invasion, *T. nativa* (23.7 specimens) is significantly inferior to *T. spiralis* (103 specimens).

Rats infected by Aira*s et al*. [18] exhibited infectivity 5–6 weeks after infection. After oral inoculation, their data revealed that the mass of muscular *T. spiralis* larvae was 57 times greater than that of *T. nativa* [19]. Sukura *et al*. [22] demonstrated that infection rates in raccoon dogs 3–6 weeks after infection were comparable for both parasites: An average of 320 larvae/g of tissue (92 muscle larvae) in *T. spiralis-*infected animals and 375 larvae/g of tissue (173 muscle larvae) in the *T. nativa-*infected group. In addition, a greater inflammatory response was observed near *T. nativa* capsules [23]. The literature shows that the immune response of the body depends on numerous factors, such as the type of organism [24], the pH [25], the type of *Trichinella* (encapsulated/non-encapsulated) [26], and the route of infection (intravenous/oral) [27].

**Figure-3:**
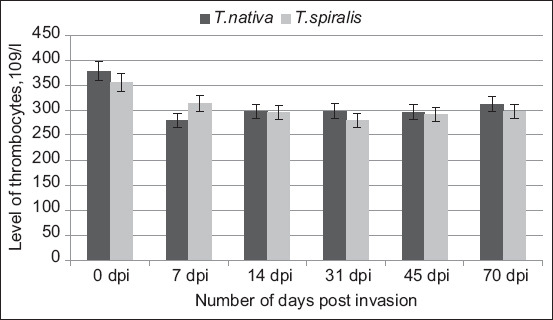
Thrombocytes level in rabbits infected with *Trichinella nativa* and *Trichinella spiralis* isolates.

As demonstrated by infections with both *T. nativa* and *T. spiralis*, a decrease in the number of thrombocytes in the blood can serve as a secondary indicator of invasion (Figure-3).

In both instances, the results revealed a decline in the thrombocyte count during infection. Moreover, in *T. nativa* infection, the platelet count was 26% lower on day 7 than it was on day 0. The number continued to increase until day 70. This phenomenon may be caused by the parasite larvae’s secretions, which reduce blood viscosity to move through the bloodstream and invade muscle tissue. On day 31, the level of thrombocytes in *T. spiralis* was 22% lower than that of the control. In both situations, the marker level fluctuates within a 17% range for the remaining days following the invasion. On day 70, the marker levels increased in both variants, which may indicate a reduction in the body’s immune defense. This marker is not necessary for the early diagnosis of infection; however, in conjunction with leukocyte formula and CK, it can be used to detect parasites within the body.

Thus, the level of CK [(on days 7 (18, 2%) and 31 (76%)] and neutrophils (day 7, 43%), as well as a decrease in the level of blood thrombocytes [(on days 7–70 (26%-17.5%)], could serve as the most important biochemical indicators for the early diagnosis of *T. nativa*.

In contrast to *T. nativa* infection, biochemical analyses of blood samples during *T. spiralis* infection showed excellent results. From Table-2, the most significant indicator for the early diagnosis of trichinellosis in blood biochemistry is an increase in neutrophil and lymphocyte levels by 26.7% and 20%, respectively, compared to the control group. In contrast to *T. nativa*, *T. spiralis* infection does not produce a robust immune response.

The value for CK on day 7 was 4.8% higher in *T. spiralis* than in *T. nativa*, which was 18.2% higher. A 26.4% decrease in the blood’s color index compared to practically stable levels of hemoglobin and erythrocytes, as well as a decrease in thrombocyte levels, may be an additional factor in the early detection of the parasite.

As an indicator of the number of newly formed blood cells, the hematocrit value changes significantly throughout the duration of *T. spiralis* infection, reaching up to 25% by day 70. Possibly because of the 14% decrease in leukocytes and the 12% decrease in thrombocytes, which affected the overall hematocrit level. Minor fluctuations in erythrocyte levels were not considered significant.

Creatine kinase levels differ significantly between *T. spiralis* and *T. nativa* infections. In this instance, the change in CK levels was deemed insignificant. There are no specific markers for the diagnosis of the intestinal and larval stages of *T. spiralis* infection. This may be due to the nonspecific immune response of rabbits to *T. nativa* infection compared to *T. spiralis* infection. When various animal species (carnivores and non-carnivores) are infected, the body’s response to infection is individualistic and largely dependent on the initial state of the animal’s immune system [28]. By day 70, the animals’ lymphocyte and CK levels began to gradually return to normal.

Damage to the host tissue is caused directly by the parasite and indirectly by the presence of inflammatory cells, which, when activated, generate high levels of reactive oxygen species and other free radicals [29]. *Trichinella spiralis* infection is accompanied by a stronger inflammatory response than infection with other *Trichinella* species.

Thus, the principal biochemical markers of early diagnosis of *T. spiralis* can be the level of neutrophils [(on days 7 (26.7%) and 31 (39.4%)] and lymphocytes (on day 7.20%), a decrease in the level of thrombocytes in the blood in both cases of infection (day 7–70 [12.4–21.6%]), and a decrease in the total number of formed cells in the blood (hematocrit).

Experimentally, biochemical parameters in rabbits infected with *T. nativa* and *T. spiralis* were significantly altered compared to the control group when analyzed using biochemical parameters. Since such markers for rabbits had not previously been published, it was not possible to confirm the data via the published literature.

In addition to hematology parameters, serological parameters were examined using ELISA following the invasion of ES-Ag. Blood sera were collected on days 7, 14, 31, 45, and 70; Table-4 displays the results of the studies.

**Table-4:** Antibody titer of rabbits infected with *Trichinella* larvae against ES-Ag of *T. nativa* and *T. spiralis* in ELISA.

Inventory/number of rabbits	Days after infestation of animals					Mean antibody titers

	7 days	14 days	31 days	45 days	70 days	
Antibody titers against ES-Ag *T. native*						
1	1:200	1:800	1:1600	1:3200	1:1600	1:1060 (+70.5; −41.3)
2	1:400	1:1600	1:3200	1:12800	1:12800	1:3200 (+94.5; −48.5)
3	1: 400	1:800	1:1600	1:6400	1:3200	1:1600 (+70.5; −41.3)
Antibody titers against ES-Ag *T. spiralis*						
4	1:400	1:1600	1:3200	1:6400	1:3200	1:2110 (+70.5; −41.3)
5	1:800	1:3200	1:6400	1:12800	1:6400	1:4220 (+70.5; −41.3)
6	1: 800	1:1600	1:6400	1:25600	1:12800	1:4850 (+94.5; −48.5)
Control group						
7	PO	PO	PO	PO	PO	-
8	PO	PO	PO	PO	PO	-
9	PO	PO	PO	PO	PO	-

ES-Ag=Excretory-secretory antigens, *T. native=Trichinella native, T. spiralis=Trichinella spiralis*, ELISA=Enzyme-linked immunosorbent assay

In subsequent use of the ES-Ag as the primary component in ELISA to diagnose trichinellosis, we observed antibodies in the blood serum of infected animals as early as day 7. However, in rabbits infected with *T. nativa*, the immune response was significantly lower than in rabbits infected with *T. spiralis:* 1:320 and 1:650. Due to the possibility of a false-positive result, it is difficult to draw definitive conclusions regarding the presence of a disease based on such titers. In contrast to the biochemical parameters of the blood on day 7, which indicated an inflammatory process due to the parasite invasion, these data do not support an inflammatory response. The reaction was more pronounced on day 7 of *T. spiralis* infection, but both results can be considered false positives. On day 14 after infection, the dynamics clearly demonstrated the presence of an immune response in both cases of infection. On day 31, the average titers for *T. nativa* and *T. spiralis* were respectively 1:6400 and 1:12400. On day 70, the number of antibodies decreased in both cases, which corresponds with the general biochemical blood test results.

The OD data from ELISA tests using *T. spiralis* and *T. nativa* ES-Ag to detect the initial signs of infection on day 7. These parameters were considered positive because their average values were four to five times higher than those of the negative control. Optical density increases up to 45 days after infection, but as with all hematological study markers, it decreases by day 70 (Table-5). On days 7, 14, and 31, the optical binding multiplicity was 5.5, 6.1, and 7.8 for *T. spiralis*, while it was 4.3, 5.2, and 7.2 for *T. nativa*. By day 45, the activity markers were nearly identical. At an early stage of infection, both parasites can be identified through a comprehensive examination of blood parameters, antibody production level, and their activity.

**Table-5:** Kinetics (OD) of IgG antibody response against ES-Ag of *T. spiralis* (n = 3) and *T. nativa* (n = 3) in ELISA.

Days	*T. spiralis*			*T. nativa*		
	
	OD	Negative control	P/P	OD of rabbits	Negative control	P/P
0	0.069 ± 0.01	0.069 ± 0.1	1.0	0.069 ± 0.01	0.071 ± 0.1	1.01
7	0.561 ± 0.02	0.101 ± 0.01	5.5	0.489 ± 0.02	0.112 ± 0.01	4.3
14	0.722 ± 0.04	0.119 ± 0.2	6.1	0.577 ± 0.04	0.111 ± 0.2	5.2
31	1.033 ± 0.05	0.132 ± 0.02	7.8	0.993 ± 0.02	0.137 ± 0.02	7.2
45	1.354 ± 0.37	0.122 ± 0.02	11	1.282 ± 0.35	0.114 ± 0.01	11.2
70	1.192 ± 0.2	0.129 ± 0.01	9.2	1.153 ± 0.33	0.125 ± 0.01	9.2

ES-Ag=Excretory-secretory antigens, *T. native=Trichinella native, T. spiralis=Trichinella spiralis*, ELISA=Enzyme-linked immunosorbent assay, OD=Optical density, IgG=Immunoglobulin G, P/P ≥ 2 – positive result, P/P ≤ 2 – negative result.

Based on serological, biochemical, and hematological studies, it can be concluded that early diagnosis of trichinellosis is possible, as demonstrated using experimentally infected rabbits. However, in this instance, it is necessary to consider the dose administered to the animals, which, in the case of a natural infection, can vary greatly and result in various body invasion reactions. The study of hematological and serological changes in the body in response to an invasion will permit the prediction of early parasite detection.

## Conclusion

Using biochemical and hematological markers for an early diagnosis of trichinellosis are seldom done today. The most crucial stage of a parasitic infestation is the initial weeks following infection. It is plausible to diagnose a possible *Trichinella* infection at these stages. In this study, we analyzed in-depth blood samples from rabbits infected with *T. spiralis* and *T. nativa* isolates and suffering from trichinellosis. The results indicate a systemic change in hematological blood parameters as early as 7 days after the parasite invasion. In addition, with *T. nativa* invasion, CK levels were found to be more accurate for early diagnosis, whereas with *T. spiralis* invasion, an increase in neutrophil and lymphocyte levels can be used for diagnosis 7 days after the invasion. In both cases of infection, the reduction in thrombocyte count (12.4–26.2%) was also an important indicator. Using the ES-Ag of the same *Trichinella* isolates, the ELISA method can detect infection as early as day 14 when using the ES-Ag. The results may permit the detection of infection on day 7, allowing for more effective treatment before encapsulation in tissues.

## Authors’ Contributions

OSA, AAI, and AMG: Drafted the manuscript and carried out the sample collection. LAL, ASS, and ZZA: Designed the study. AKZ, ZKB, and FSZ: Carried out laboratory analyses. All authors have read and approved the final manuscript.
